# VarSim: A Python Toolkit for HGVS Variant Description Validation, Normalization, and Representation Conversion

**DOI:** 10.1155/humu/6006096

**Published:** 2026-10-09

**Authors:** Liu Sun, Rongshu Wang, Jian Yang

**Affiliations:** ^1^ Department of Computer Science School of Engineering Yuxi Normal University Yuxi China yxnu.net; ^2^ Yunnan Key Laboratory of Smart City in Cyberspace Security Yuxi Normal University Yuxi China yxnu.net

**Keywords:** bioinformatics, HGVS nomenclature, MANE, variant normalization, variant validation

## Abstract

Accurate and reproducible representation of genetic variants is essential for clinical genomics, variant databases, and genomic research. The Human Genome Variation Society (HGVS) recommendations provide a standardized framework for describing sequence variants, but practical HGVS processing commonly involves multiple interdependent tasks, including parsing, validation, normalization, coordinate transformation, protein consequence inference, and conversion between variant representation formats. We developed VarSim, a lightweight Python toolkit that integrates these operations within a reference‐aware workflow based on version‐pinned Matched Annotation from NCBI and EMBL‐EBI (MANE) transcripts retrieved from NCBI Entrez. VarSim provides programmatic functions for HGVS parsing, syntactic and semantic validation, normalization, sequence extraction, backtranslation, coordinate mapping, protein consequence prediction, genome‐assembly liftover, and conversion between HGVS, VCF, and SPDI representations. We benchmarked the toolkit using 1458 HGVS descriptions across five MANE transcripts, comprising 500 coding single‐nucleotide variants, 250 frameshift insertions/deletions, and 708 intronic splice‐site substitutions. All 1458 descriptions passed VarSim syntactic validation, and all 750 coding descriptions passed semantic validation against the corresponding reference sequences. VariantValidator accepted all 1458 descriptions, whereas the Mutalyzer normalization endpoint processed the 750 coding descriptions. VarSim normalization agreed with Mutalyzer for all 750 coding variants and with VariantValidator for 749 of 750 variants. Protein consequence predictions agreed with VariantValidator for all 500 coding single‐nucleotide variants. Format conversion between HGVS, VCF, and SPDI was exact for the tested variants, whereas transcript‐to‐genome projection showed substantial discrepancies relative to VariantValidator and therefore remains a limitation of the current implementation. VarSim provides a complementary, programmatic framework for reproducible HGVS processing and variant representation interoperability.

## 1. Introduction

Standardized representation of sequence variants is fundamental to the interpretation, reporting, and exchange of genetic information. In clinical and research settings, a variant description must not only identify the sequence change but also provide sufficient reference context to ensure that the description is unambiguous and reproducible. The Human Genome Variation Society (HGVS) recommendations provide a widely adopted framework for describing variants at the DNA, RNA, and protein levels [1, 2]. Accurate implementation of these recommendations is therefore important for clinical reporting, database curation, genomic analysis, and communication of variant findings.

Computational processing of HGVS descriptions involves several related but distinct operations. A variant description may first need to be parsed into a structured representation and validated against an appropriate reference sequence. Equivalent sequence changes may then require normalization according to HGVS rules, including application of the 3 ^′^ rule and representation of insertions as duplications where appropriate. Downstream analyses may require transformation between transcript and genomic coordinates, inference of protein consequences, or conversion between HGVS and other variant representation systems such as VCF and SPDI. These operations are dependent on reference‐sequence selection and accession version, making explicit reference context an important component of reproducible variant processing.

Several established resources provide complementary functionality for HGVS processing. Mutalyzer provides HGVS validation and normalization services, whereas VariantValidator supports validation, mapping, and formatting of sequence variation descriptions [3, 4]. The hgvs Python package provides a programmatic framework for manipulating sequence variants according to HGVS nomenclature [5], and LOVD3 provides infrastructure for variant collection and sharing [6]. Although these resources address different aspects of variant representation, workflows that combine multiple HGVS operations may require several interfaces, reference resources, or local database configurations.

The availability of the MANE transcript set provides an additional opportunity to establish a consistent reference context for computational variant processing. MANE transcripts are jointly developed by NCBI and EMBL‐EBI to provide harmonized transcript representations for clinical genomics and research [7]. Using version‐pinned MANE accessions can reduce ambiguity arising from transcript selection and provide an explicit reference sequence for downstream operations.

To address the need for an integrated and lightweight programmatic workflow, we developed VarSim, a Python toolkit for HGVS variant description processing. VarSim combines parsing, syntactic and semantic validation, normalization, sequence extraction, backtranslation, coordinate transformation, protein consequence inference, genome‐assembly liftover, and representation conversion. The toolkit retrieves reference records dynamically from NCBI Entrez and uses versioned MANE accessions as the primary reference context. Here, we describe the implementation of VarSim and evaluate its performance against established HGVS validation resources using a benchmark set of 1458 HGVS descriptions.

## 2. Materials and Methods

### 2.1. Software Architecture

VarSim is implemented in Python (≥ 3.8) and is designed as a lightweight package with a minimal software dependency structure. Biopython is used for sequence‐related operations [8]. The toolkit represents parsed HGVS descriptions as structured HGVS tag objects and exposes programmatic functions for validation, normalization, coordinate transformation, sequence extraction, translation, backtranslation, and representation conversion (Table 1).

**Table 1:** VarSim modules and HGVS software functionality.

Functionality	Module/API	What it does
Parsing	parse()	HGVS descriptions → structured HGVSTag object model (c. p. g. n. m.)
Validation	is_valid_syntax(); is_valid()	Syntactic form; semantic checks against reference sequences
Normalization	normalize()	3 ^′^ shift (del/dup), ins → dup, allele minimization, ranges
Conversion	hgvs_to_vcf(); hgvs_to_spdi(); vcf_to_hgvs(); spdi_to_hgvs()	HGVS ↔ VCF/SPDI
Extraction	extract(); extract_with_positions()	Minimal HGVS description from reference vs observed sequence
Backtranslation	backtranslate(); backtranslate_protein()	c. candidates for an observed p. change
Liftover	liftover_g_to_assembly(); liftover_transcript()	GRCh37 ↔ GRCh38 via NCBI Remap
Transcription	c_to_g(); g_to_c()	Transcript (c.) ↔ genome (g.) coordinate mapping
Translation	translate_variant(); get_protein_effect()	p. consequence inference for c. variants

*Note:* API names are reproduced from the manuscript and refer to the corresponding VarSim functions or modules.

The overall workflow is illustrated in Figure 1. Reference sequences are retrieved according to the requested gene or accession, HGVS descriptions are parsed and validated, and validated descriptions can subsequently be normalized or transformed between sequence and representation systems.

**Figure 1:**
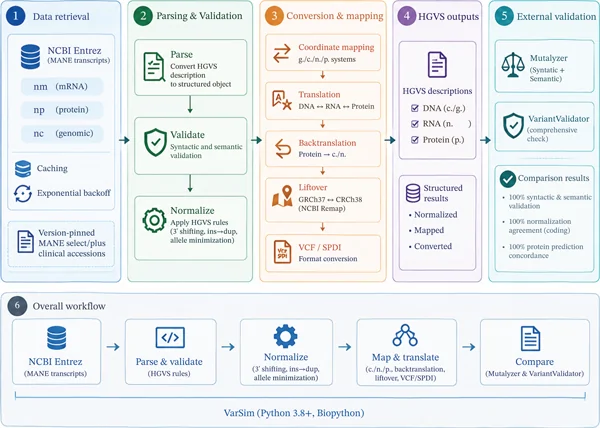
Overview of the VarSim workflow. Version‐pinned MANE transcripts are retrieved from NCBI Entrez and used as the reference context for HGVS parsing, validation, and normalization. Validated descriptions can be transformed across transcript, genomic, and protein representations and converted between HGVS, VCF, and SPDI representations. VarSim outputs were benchmarked against Mutalyzer and VariantValidator using 1458 HGVS descriptions across five MANE transcripts.

### 2.2. Reference Sequence Retrieval and MANE Transcript Selection

VarSim retrieves nucleotide, protein, and genomic reference records through the NCBI Entrez API. For gene‐based operations, the toolkit prioritizes MANE Select and MANE Plus Clinical accessions. Reference accessions are retained with their version identifiers throughout the processing workflow.

For example, the HBB MANE transcript is represented by NM_000518.5. Versioned accessions provide an explicit reference sequence context and allow results to be reproduced using the same sequence version. The validation and normalization functions also accept user‐supplied reference sequences, allowing processing against transcript or protein records outside the MANE set when required.

To reduce repeated external requests, VarSim implements local LRU caching. An exponential back off strategy is used to handle transient failures and rate limiting during repeated NCBI requests.

### 2.3. HGVS Parsing and Validation

VarSim parses HGVS descriptions into structured representations covering the principal HGVS sequence prefixes, including c., p., g., n., and m. Syntactic validation evaluates the formal structure of the description, including nucleotide and amino‐acid notation, coordinate syntax, and range ordering.

Semantic validation compares the reference allele specified by the HGVS description with the corresponding nucleotide or amino‐acid sequence in the supplied reference record. In the benchmark described below, semantic validation was evaluated for coding descriptions against the corresponding reference CDS.

### 2.4. HGVS Normalization

Normalization aims to represent equivalent sequence changes according to HGVS recommendations while preserving the underlying sequence alteration. VarSim implements the HGVS 3 ^′^ rule for deletions and duplications and does not apply positional shifting to substitutions. Insertions that duplicate adjacent reference sequence can be represented as duplications, with the resulting duplication positioned according to the 3 ^′^ rule.

The normalization module also performs allele minimization and coordinate‐range normalization where applicable. Because normalization depends on the reference sequence, the reference accession and version are retained as part of the processing context

### 2.5. Sequence Extraction and Backtranslation

The extract () functionality compares reference and observed sequences and derives a minimal HGVS representation of the sequence difference. An extended extraction function also reports the corresponding positions used during the comparison.

Backtranslation addresses the inverse relationship between protein and coding‐sequence representations. Given a protein‐level change, VarSim enumerates candidate coding SNVs that could produce the specified amino‐acid substitution and evaluates the candidates against the corresponding reference CDS.

### 2.6. Coordinate Mapping and Protein Consequence Prediction

VarSim provides functions for transforming coding coordinates between transcript and genomic representations. Transcript‐to‐genome mapping is based on exon information obtained from the corresponding RefSeq transcript record. The reverse transformation is also supported.

For coding variants, the translation engine determines the predicted protein consequence from the reference and altered CDS sequences. The supported consequence categories include synonymous, missense, nonsense, stop‐loss, extension, and frameshift changes.

### 2.7. HGVS, VCF, and SPDI Conversion

VarSim supports conversion between HGVS and commonly used variant representation formats. HGVS descriptions can be converted to VCF and SPDI representations, and VCF or SPDI records can subsequently be converted back to HGVS descriptions.

These functions were evaluated using round‐trip conversion tests to determine whether the original sequence representation was preserved.

### 2.8. Genome‐Assembly Liftover

VarSim provides helper functions for genome‐assembly coordinate conversion, including GRCh37‐to‐GRCh38 and GRCh38‐to‐GRCh37 operations. These functions use the NCBI Remap service. Because the operation depends on an external service, its availability and output are dependent on the response returned by NCBI.

### 2.9. Benchmark Dataset

The benchmark dataset comprised 1458 HGVS descriptions distributed across five MANE transcripts: HBB (NM_000518.5), G6PD (NM_001360016.2), TP53 (NM_000546.6), CFTR (NM_000492.4), and GNAL (NM_182978.4). The dataset included 500 coding SNVs, 250 one‐base frameshift insertions/deletions, and 708 intronic splice‐site substitutions.

The benchmark was designed to evaluate representative HGVS processing tasks involving coding substitutions, indels, and intronic splice‐site descriptions across transcripts with different sequence contexts.

### 2.10. Comparative Validation

Each benchmark description was processed by VarSim and submitted to VariantValidator and the Mutalyzer normalization endpoint. For each resource, acceptance or error status and normalized output were recorded.

Syntactic and semantic validation were evaluated using VarSim. Acceptance by VariantValidator and Mutalyzer was recorded independently. Normalized outputs were compared when both tools returned a result for the same description.

Protein consequence predictions were compared with VariantValidator for the 500 coding SNVs. Transcript‐to‐genome projections were independently compared with VariantValidator using 50 coding SNVs, comprising 10 variants per gene.

Additional tests evaluated backtranslation, sequence extraction, HGVS‐to‐VCF conversion, HGVS‐to‐SPDI conversion, and round‐trip conversion between the different representations.

### 2.11. Performance Evaluation

Runtime was measured on a commodity laptop under the same software environment used for the benchmark. Parser and validation performance was evaluated for all 1458 descriptions, normalization performance for the 750 coding variants, and protein consequence inference for the 500 coding SNVs. Runtime was evaluated both with and without cached reference records where applicable.

## 3. Results

### 3.1. HGVS Validation

VarSim successfully parsed and syntactically validated all 1,458 descriptions in the benchmark dataset (1458/1458; 100%). All 750 coding descriptions also passed semantic validation against the corresponding reference sequences.

VariantValidator accepted all 1458 descriptions. The Mutalyzer normalization endpoint processed all 750 coding descriptions but returned errors for the 708 intronic splice‐site descriptions in the benchmark. These descriptions represented c.+1/+2 and c.−1/−2 positions for which the selected Mutalyzer normalization endpoint requires additional selector context. The endpoint behavior was therefore treated separately from the validity of the descriptions themselves.

### 3.2. Normalization Concordance

For the 750 coding variants processed by Mutalyzer, VarSim produced normalized descriptions identical to Mutalyzer in all cases (750/750; 100%) (Table 2).

**Table 2:** Cross‐validation of VarSim against Mutalyzer and VariantValidator on 1458 HGVS descriptions.

Gene	MANE transcript	Tested	Syntactically valid (VarSim)	Semantically valid (VarSim)	Accepted by Mutalyzer	Accepted by VariantValidator	Normalization identical (VarSim–Mutalyzer)
HBB	NM_000518.5	174	174	150	150	174	150
G6PD	NM_001360016.2	282	282	150	150	282	150
TP53	NM_000546.6	258	258	150	150	258	150
CFTR	NM_000492.4	462	462	150	150	462	149
GNAL	NM_182978.4	282	282	150	150	282	150
Total	—	1458	1458	750	750	1458	749

*Note:* Syntactic validation refers to successful parsing of the HGVS description. Semantic validation additionally checks the described reference allele against the corresponding reference sequence. Mutalyzer acceptance reflects the selected endpoint and its handling of the submitted descriptions; the 708 intronic splice‐site descriptions were not processed by that endpoint because selector‐context requirements were not satisfied. The single VarSim–VariantValidator normalization discrepancy was CFTR c.3962_3963insG, for which VarSim and Mutalyzer returned c.3964dup whereas VariantValidator returned c.3963+1dup. These descriptions represent an equivalent sequence event at an exon boundary.

Comparison with VariantValidator showed agreement for 749 of 750 coding variants (99.9%) (Table 2). The single difference involved CFTR c.3962_3963insG. All three systems represented the event as a duplication, but VarSim and Mutalyzer reported c.3964dup, whereas VariantValidator reported c.3963+1dup. These descriptions represent the same sequence event at an exon boundary but use different coordinate contexts.

Additional tests in repetitive HBB sequence contexts demonstrated the expected 3 ^′^ shifting of deletions and duplications. For example, NM_000518.5.41del was normalized to c.43del, and c.47del was normalized to c.50del. Insertions duplicating adjacent sequence were converted to duplications and placed at the corresponding 3 ^′^‐most position. Substitutions were not shifted.

These results indicate that VarSim reproduces the normalization behavior of established HGVS resources for the evaluated coding variants while also illustrating that equivalent sequence events may occasionally have alternative coordinate representations at transcript boundaries.

### 3.3. Protein Consequence Prediction

VarSim protein consequence predictions agreed with VariantValidator for all 500 coding SNVs evaluated (500/500; 100%).

The tested variants included synonymous and amino‐acid‐altering substitutions, and the translation engine correctly generated the corresponding protein‐level consequences from the reference and altered coding sequences.

### 3.4. Backtranslation and Sequence Extraction

Backtranslation was evaluated using 23 missense substitutions. In all cases, the corresponding coding SNV was recovered among the candidate c.HGVS descriptions generated from the protein‐level change.

The sequence extraction function was evaluated using 100 reference/observed sequence pairs, comprising 50 SNVs and 50 one‐base deletions. The function generated the expected minimal HGVS descriptions for all tested pairs. Deletions occurring within homopolymeric sequences were represented at their normalized 3 ^′^‐shifted positions.

### 3.5. Representation Conversion

HGVS‐to‐VCF conversion was evaluated for 50 genomic descriptions and reproduced the corresponding VariantValidator VCF position, reference allele, and alternate allele in all tested cases.

HGVS‐to‐SPDI, VCF‐to‐HGVS, and SPDI‐to‐HGVS conversions also produced exact round‐trip results for the evaluated variants. These findings indicate that the tested conversion functions preserved the represented sequence change across the evaluated formats.

### 3.6. Transcript‐to‐Genome Projection

Transcript‐to‐genome projection showed substantially lower concordance with VariantValidator than the other evaluated functions. Fifty coding SNVs were evaluated, with 10 variants selected from each of the five benchmark genes. None of the VarSim projections matched the corresponding VariantValidator primary‐assembly locus exactly. For example, CFTR c.325T>A was projected by VarSim as NC_000007.14.117480419T>A, whereas VariantValidator reported NC_000007.14.117530950T>A. Additional discrepancies were observed for minus‐strand transcripts.

These findings indicate that the current transcript‐to‐genome projection implementation should not be considered an authoritative source of genomic coordinates. Genomic coordinates generated by this function should therefore be independently checked using an established validation resource.

### 3.7. Genome‐Assembly Liftover

The GRCh37/GRCh38 liftover functions depend on the NCBI Remap service. During testing, when the external service was unavailable, the functions returned the input unchanged. Accordingly, liftover should be regarded as a service‐dependent operation and its results should be checked when external service failures occur.

### 3.8. Computational Performance

VarSim processed all 1458 benchmark descriptions for parsing and validation in less than 0.1 s, corresponding to approximately 15,000 descriptions/s under the tested conditions. Normalization of the 750 coding variants also required less than 0.1 s.

Protein consequence inference for the 500 coding SNVs required approximately 7 s when reference records were retrieved remotely and less than 1 s when the relevant records were already cached. Thus, under the tested conditions, external reference retrieval contributed substantially to runtime for operations requiring uncached records.

### 3.9. Application Example

The complete workflow was illustrated using the classical sickle‐cell variant HBB NM_000518.5.20A>T, corresponding to p.Glu7Val. VarSim parsed the HGVS description into a structured representation, validated it against the reference CDS, normalized the description, inferred the protein consequence, and identified the corresponding coding SNV during backtranslation. The same workflow also supported conversion of the corresponding genomic representation to VCF.

## 4. Discussion

Accurate and reproducible variant description is fundamental to the interpretation, reporting, and exchange of genetic information. Although HGVS nomenclature provides a standardized framework for representing sequence variants, practical implementation of HGVS‐compliant workflows often requires several distinct operations, including parsing, validation, normalization, coordinate conversion, and prediction of protein consequences. VarSim was developed to integrate these operations into a lightweight Python‐based workflow anchored to version‐pinned MANE transcripts. In the present benchmark, all 1458 tested descriptions passed VarSim′s syntactic validation, all 750 coding descriptions passed semantic validation against the corresponding reference sequences, and the resulting normalization and protein‐effect predictions showed high concordance with established reference services. These results support the utility of VarSim as a programmatic framework for consistent processing of HGVS descriptions rather than as a replacement for existing specialist validation services.

A major strength of VarSim is the integration of reference‐sequence retrieval with HGVS description processing. By resolving versioned MANE transcripts through NCBI Entrez, VarSim provides an explicit reference context for variant validation and normalization. This is particularly relevant to clinical and research workflows in which the same variant may otherwise be represented differently depending on transcript selection or reference‐sequence version. The use of version‐pinned accessions also provides a practical basis for reproducibility when variant descriptions are generated or reprocessed over time. In contrast to workflows that require users to install and maintain large local sequence resources, VarSim retrieves the required reference records dynamically and uses caching to reduce repeated queries. This architecture may therefore be useful for lightweight scripting, database curation, automated quality control, and cloud‐based workflows in which maintaining a local reference infrastructure is undesirable.

The benchmarking results further demonstrate that VarSim reproduces key aspects of established HGVS processing. Normalization agreed with Mutalyzer for all 750 coding variants processed by the Mutalyzer normalization endpoint, supporting the implementation of HGVS normalization rules such as 3 ^′^ shifting, insertion‐to‐duplication conversion, and allele minimization. The single normalization discrepancy observed between VarSim and VariantValidator represented alternative HGVS formulations of the same sequence event rather than a difference in the underlying variant. This observation is important because apparently different HGVS strings do not necessarily indicate biologically different variants; normalization must therefore be evaluated in the context of sequence equivalence and reference‐coordinate conventions. Similarly, VarSim′s protein‐effect predictions agreed with VariantValidator for all 500 coding SNVs evaluated, indicating that the integrated translation functionality can provide consistent consequences for coding substitutions. Together, these findings suggest that VarSim can serve as a practical intermediate layer between raw variant descriptions and downstream annotation or interpretation workflows.

An additional feature of VarSim is its support for bidirectional and cross‐representation operations. Variant descriptions can be transformed between transcript, protein, and genomic coordinate systems, and between HGVS, VCF, and SPDI representations. Such interoperability is increasingly important because variant data are routinely exchanged between clinical databases, variant repositories, analysis pipelines, and computational tools that use different representation schemes. The successful round‐trip testing of HGVS, VCF, and SPDI conversions provides evidence that these functions can support programmatic data exchange while preserving the represented sequence change. Backtranslation and sequence‐based extraction further extend the toolkit beyond conventional HGVS validation by allowing users to explore the relationship between sequence‐level changes and their corresponding variant descriptions. These capabilities may be particularly useful for systematic variant enumeration and in‐silico mutagenesis, where large numbers of standardized variant descriptions must be generated and subsequently checked.

The results also highlight an important distinction between HGVS description validation and genomic coordinate projection. VarSim performed consistently for parsing, validation, coding‐variant normalization, and protein‐effect prediction, but transcript‐to‐genome projection showed substantial discrepancies relative to VariantValidator in the tested set. None of the 50 evaluated SNV projections matched the corresponding VariantValidator primary‐assembly loci exactly, including examples involving intron‐spanning coordinates and transcripts on the minus strand. This finding indicates that genomic projection requires particularly careful handling of transcript structure, strand orientation, and genomic anchoring. Consequently, VarSim‐generated genomic coordinates should not currently be considered equivalent to independently validated genomic loci and should be cross‐checked with an established external validator when precise genomic placement is required.

Several other limitations should be considered. VarSim depends on real‐time NCBI Entrez access and is therefore sensitive to network availability, external service performance, and rate limits. Caching and retry mechanisms mitigate these constraints but do not eliminate the dependency on external reference resources. Similarly, genome‐assembly liftover depends on the availability of the NCBI Remap service and should therefore be regarded as a service‐dependent operation. In addition, the present benchmark included 1458 HGVS descriptions across five MANE transcripts. Although the dataset covered coding, intronic splice‐site, and frameshift descriptions and included multiple HGVS processing tasks, it does not capture the full diversity of variant representations encountered across genes, transcript architectures, genomic regions, and reference assemblies. Broader evaluation using larger and more diverse datasets will therefore be important for defining the generalizability of VarSim across clinical and research applications.

Overall, VarSim provides a compact framework for integrating multiple stages of HGVS variant description processing while maintaining an explicit reference‐sequence context. Its strongest current applications are likely to be automated parsing and validation, coding‐variant normalization, protein‐effect inference, standardized representation conversion, and generation or quality control of large variant datasets. The benchmarking results demonstrate high concordance with established validation resources for these functions, while the identified limitations emphasize that genomic coordinate projection and externally dependent operations require additional verification. Rather than replacing specialized resources such as Mutalyzer or VariantValidator, VarSim is best positioned as a complementary, programmable component that facilitates reproducible HGVS processing within broader bioinformatic and variant‐interpretation workflows.

## 5. Conclusions

VarSim provides a lightweight Python framework for programmatic processing of HGVS variant descriptions, including parsing, syntactic and semantic validation, normalization, protein consequence inference, sequence extraction, backtranslation, representation conversion, and genome‐assembly liftover. In a benchmark of 1458 descriptions across five MANE transcripts, all tested descriptions passed VarSim syntactic validation; all coding descriptions passed semantic validation; normalization agreed with Mutalyzer for all 750 coding variants evaluated by that endpoint; and protein consequence predictions agreed with VariantValidator for all 500 coding SNVs. HGVS, VCF, and SPDI conversion also showed exact results in the tested cases.

At the same time, transcript‐to‐genome projection showed substantial discrepancies relative to VariantValidator, indicating that this functionality requires independent verification and further development. VarSim should therefore be considered a complementary computational component for HGVS processing and variant‐data interoperability rather than a replacement for established specialist validation services.

## Funding

This work was supported by the National Natural Science Foundation of China (Grant No. 62266048), the Yunnan Key Laboratory of Smart City in Cyberspace Security (Grant No. 202105AG070010), and Yunnan Province Expert Workstations (Grant No. 202305AF150036).

## Conflicts of Interest

The authors declare no conflicts of interest.

## Data Availability

VarSim is distributed under the open‐source MIT License. The Python library can be installed directly from PyPI (https://pypi.org/project/varsim), and the full source code is actively maintained at GitHub (https://github.com/liu-sun/VarSim). The datasets, benchmarking scripts, and validation results underlying this study are archived with the source repository (benchmarks/).
